# Acute pain and side effects after tramadol in breast cancer patients: results of a prospective double-blind randomized study

**DOI:** 10.1038/s41598-020-75961-2

**Published:** 2020-10-30

**Authors:** Nikola Besic, Jaka Smrekar, Branka Strazisar

**Affiliations:** 1 Department of Surgical Oncology, Institute of Oncology, Zaloska 2, 1000 Ljubljana, Slovenia; 2 Faculty of Mathematics and Physics, University of Ljubljana, 1000 Ljubljana, Slovenia; 3 Department of Anesthesiology, Institute of Oncology, Zaloska 2, 1000 Ljubljana, Slovenia

**Keywords:** Health care, Medical research, Oncology, Signs and symptoms

## Abstract

The objective of this study was to evaluate the severity of acute pain and side effects in breast cancer patients postoperatively treated with two regimens of tramadol with paracetamol in a prospective double-blind study. Altogether 117 breast cancer patients who had axillary lymphadenectomy were randomized into two analgesic study groups and the analgesic treatment lasted 4 weeks. Stronger analgesia group received every 8 h 75/650 mg of tramadol with paracetamol, while weaker analgesia group received every 8 h 37.5/325 mg of tramadol with paracetamol. Patients with the higher dose of tramadol had less pain during the 1st and 4th week than patients with the lower dose. Frequency of nausea, vomiting, lymphedema or range of shoulder movement was not significantly different between the two groups of patients. Constipation was significantly more common in the group with stronger analgesia during the 2nd week in comparison to patients with weaker analgesia. The patients who were on 75/650 mg of tramadol with paracetamol had less pain in comparison to patients who were on 37.5/325 mg. Side effects were mild, but common in both groups of patients.

## Introduction

Breast surgery is known to cause severe acute postoperative pain in more than 50% of patients^1^. Adverse effects were reported even more common after axillary lymphadenectomy. Gartner et al.^2^ and Lucci et al.^3^ reported adverse effects in 70% of patients. Axillary surgery severely affects quality of life because it may have a deteriorating effect on arm morbidity, movement limitations, swelling and lymph drainage^4^.

Several non-steroidal anti-inflammatory drugs, opioids and several other pharmacological agents and regional anesthesia are used in order to decrease postoperative pain^5–7^. Tramadol is a treatment of choice for pain management after axillary lymph node dissection in breast cancer patients^8^. Tramadol is commonly used for pain treatment because it produces satisfactory analgesia against various types of pain, and it is currently approved for the treatment of moderate to severe pain^9^. Furthermore, it induces fewer side effects than classic opioids^9^. However, some patients experience insufficient pain relief or adverse events^10^. The aim of this study was to evaluate the severity of acute pain and the frequency of side effects in breast cancer patients treated with two regimens of tramadol with paracetamol after axillary lymphadenectomy.

## Methods

### Patients

A prospective double-blind randomized study included 117 breast cancer patients (median age 54 years, range 24–70 years) receiving tramadol for pain relief after axillary lymphadenectomy (Trial KCT 04/2015-doretaonko/si, EudraCT Number: 2015-000992-28, date of registration 10/06/2015). The patients were treated at the Institute of Oncology Ljubljana from 2015 to 2018. Eligibility criteria for inclusion in the study were: axillary lymphadenectomy and patient’s age from 18 to 74 years. Exclusion criteria were: concomitant breast reconstruction with a tissue expander or free-flap, hypersensitivity to the drugs used in the study, pregnancy, high anesthesia risk, severe liver or kidney disease, patients taking medicines that could affect the outcome of the treatment. Therefore, exclusion criteria were also regular use of analgesics or antidepressants, history of opioid abuse or presence of psychiatric illness (dementia, schizophrenia, manic depressive illness). Patient’s characteristics, tumor characteristics and treatment of patients are presented in Table 1.

**Table 1:** Patient’s characteristics, tumor characteristics and treatment of patients.

Characteristic	Sub-group	Stronger postoperative analgesia with tramadol and paracetamol	Weaker postoperative analgesia with tramadol and paracetamol	p-value
Number of patients		59	58	–
Age (years)—mean		55.0	53.2	0.35
Height (m)—mean		165.5	163.4	0.63
Weight (kg)—mean		73.0	73.9	0.68
Body Mass Index (kg/m^2^)—mean		27.2	27.9	0.49
ASA score	1	13	12	0.71
	2	40	43	
	3	6	3	
Concomitant diseases	Yes	47	41	0.29
	No	12	17	
Histology	Invasive ductal carcinoma	47	44	0.68
	Invasive lobular carcinoma	6	9	
	Other type of invasive carcinoma	6	5	
Tumor diameter (cm)—mean		2.88	2.95	0.79
Gradus	I	1	3	0.42
	II	20	23	
	III	38	32	
Hormone receptors	Positive	47	48	0.67
	Negative	12	10	
HER-2 positive	Yes	7	13	0.13
	No	52	45	
Mastectomy side	Left	30	32	0.57
	Right	28	26	
	Bilateral	1	0	
Breast surgical procedure and lymphadenectomy	Simultaneous	39	42	0.46
	Previous surgery	20	16	
Simultaneous breast cancer procedure (N = 81)	Mastectomy	20	28	0.19
	Breast conserving surgery	19	14	
Axillary lymphadenectomy	Left	30	32	0.57
	Right	28	26	
	Bilateral	1	0	
Number of resected lymph nodes—mean		19.6	18.8	0.57
Number of metastatic lymph nodes—mean		6.4	4.6	0.20
Chemotherapy	Yes	52	48	0.41
	No	7	10	
Neoadjuvant chemotherapy	Yes	9	17	0.07
	No	50	41	
Post-operative radiotherapy	Yes	53	52	0.97
	No	6	6	
Hormone therapy	Yes	47	48	0.67
	No	12	10	
HER-2 therapy	Yes	7	13	0.13
	No	52	45	

### Study protocol

After informed consent was obtained from the patient, the randomization was carried out before surgery by nurses at the clinical research unit of the Institute of Oncology Ljubljana.

The surgical procedure was performed during general anesthesia. The anesthetic technique for all the patients was the same. For 2 days, all patients had a continuous local infusion of 0.25% levo-bupivacaine with a fenestrated wound catheter and flow rate of 2 mL per hour as reported by Strazisar et al.^7^. The catheter was removed on the morning of the second postoperative day.

On the first postoperative day all patients received tramadol with a paracetamol dose of 37.5/325 mg every 8 h. All patients received naproxen sodium 550 mg every 12 h. On the first day after surgery, all patients received metoclopramide 10 mg every 8 hours to prevent nausea. During hospital stay a 'rescue' drug was piritramide 3 mg i.v. when VAS score was ≥ 4. In recovery room 23% of patients received a ‘rescue’ drug, after 8 h 13.6% received a ‘rescue’ drug and on the 1st postoperative day only 5% of patients received a ‘rescue’ drug.

The patients received different analgesia from the 2nd to the 29th postoperative day as already reported in our paper about chronic adverse effects after an axillary lymphadenectomy^11^. Each patient used one of two analgesic regimens. Patients were randomized into two groups: a group with weaker post-operative analgesia and a group with stronger analgesia. Neither patients nor healthcare professionals knew which patient was being treated with a lower dose and which with a higher dose of analgesia. The patients obtained packages with the medicines at the Oncology Institute's pharmacy, where once a week a pharmacist checked how many medications the patient had consumed. Each week, the patients received two packages of drugs. The first package contained pills with 37.5/325 mg of tramadol with paracetamol. The second package contained the test drugs: either placebo or 37.5/325 mg of tramadol with paracetamol. Both pills (placebo and those with 37.5/325 mg of tramadol with paracetamol) had the same appearance. Each patient had to take one pill from both packages every 8 h^11^. All patients also received twice daily naproxen sodium 550 mg and once a day pantoprazole 20 mg, for 4 weeks. In the case of severe pain, despite taking tramadol and paracetamol and naproxen sodium, patients received metamizole 500 mg (up to 4 g per day). Because of pain only one patient used a rescue drug^11^.

From the 2nd to the 29th day after surgery, all patients recorded in their diary how many medications were consumed and how many times they carried out shoulder exercises. Also, data on the severity of pain in the area of the breast and shoulder were recorded three times a day using a standard visual analogue scale (VAS) score ranging from 0 to 10. Furthermore, the occurrence of nausea, vomiting and obstipation were recorded in the diary.

The occurrence of adverse effects from the treatment, shoulder mobility and the presence of lymphedema were recorded in patients’ charts during their visit to an outpatients’ clinic every 7 days for the first 4 weeks after surgery. Each week, patients had physiotherapy with our physiotherapist, who also measured shoulder movements.

**Figure 1:**
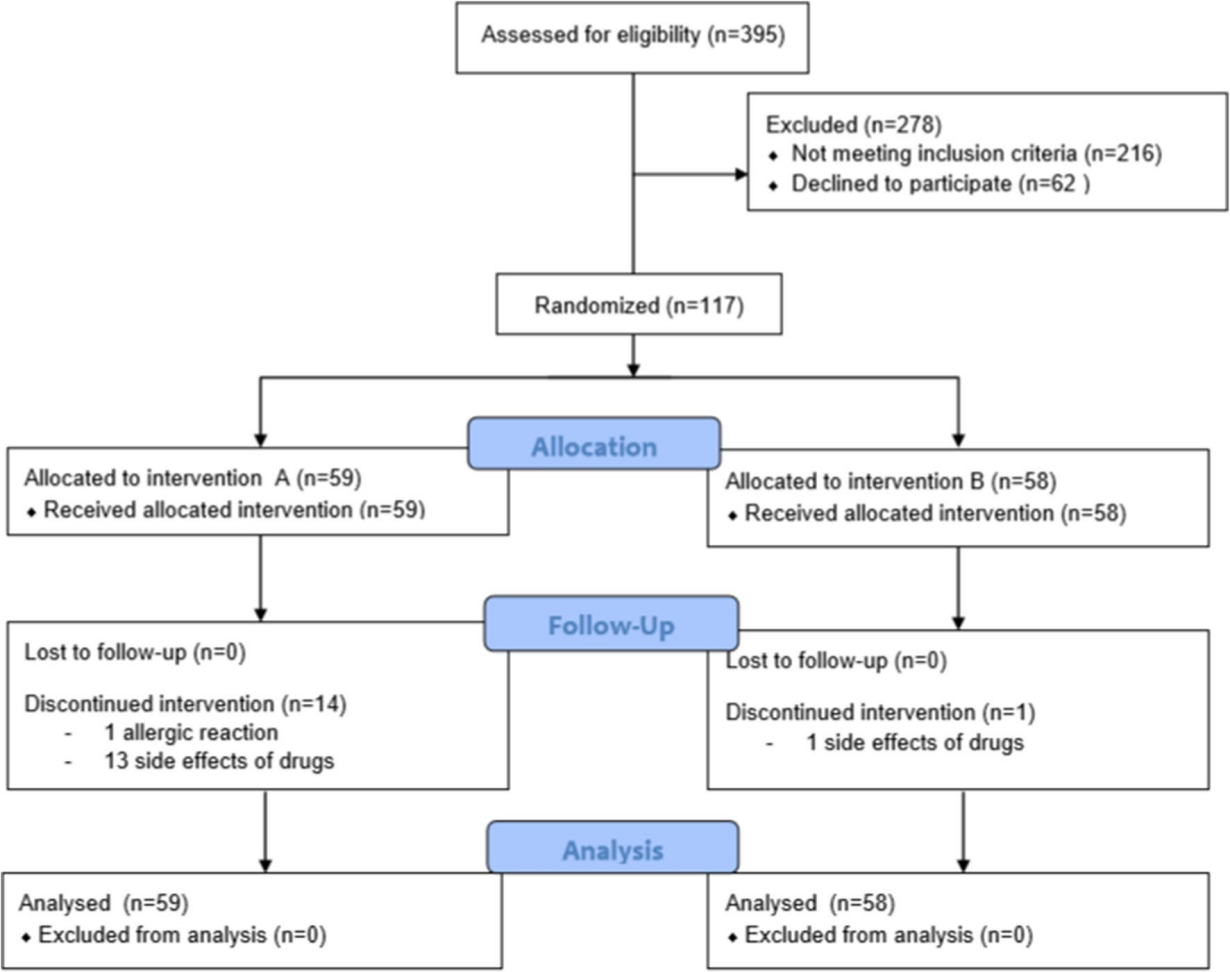
Flow diagram of our study.

Patients (N = 15) who decided to abandon the study protocol were asked to visit regularly our outpatients’ clinic once a week, and fill out the diary and all questionnaires. Figure 1 illustrates the complete patient flow.

### Statistical analysis

The sample size was calculated on the basis of our previous research on acute pain after breast cancer surgery after axillary lymph node dissection (using Cohen's d statistical analysis) as already reported in our paper about chronic adverse effects after an axillary lymphadenectomy^11^. Given the likelihood of data loss in the group with weaker analgesia of 0.10, and 0.15 in the group with stronger analgesia, 58 and 55 patients were needed to warrant a power of 0.80 at our assumptions. Our cutoff for significance was 0.05. Statistical analysis was performed on the intention-to-treat population^11^.

For statistical comparisons between groups with weaker and stronger postoperative analgesia we used standard methods for unpaired comparisons without the assumption of homoscedasticity, based on asymptotic normality due to reasonably large samples^11^. The statistical analysis was performed on the intention-to-treat sample, and all was repeated for the treatment received sample. Both analyses yielded similar results with no significant change in outcomes^11^. Statistical analysis was done by J.S. using Microsoft Excel 2016 for data manipulation.

### Ethical approval

All procedures performed in studies involving human participants were in accordance with the ethical standards of the institutional and/or national research committee and with the 1964 Helsinki declaration and its later amendments or comparable ethical standards. The study Trial KCT 04/2015-doretaonko/si, EudraCT Number: 2015-000992-28 met the guidelines of their responsible governmental agency and was reviewed and approved by The National Medical Ethics Committee of the Republic of Slovenia (Approval number 32/03/15). Our study was approved by the Institutional Review Board of the Institute of Oncology Ljubljana.

### Informed consent

Informed consent was obtained from all individual participants included in the study.

## Results

Both treatment groups (higher and lower dose) were found comparable on the basis of age, weight, ASA physical status classes, concomitant diseases, surgery, histopathology of tumor and cancer treatment (Table 1). Our patients had a high burden of disease in the axilla. The median number of positive lymph nodes in a higher dose group of analgesia and in a lower group of postoperative analgesia was even 6.4 and 4.6, respectively.

### Pain

**Figure 2:**
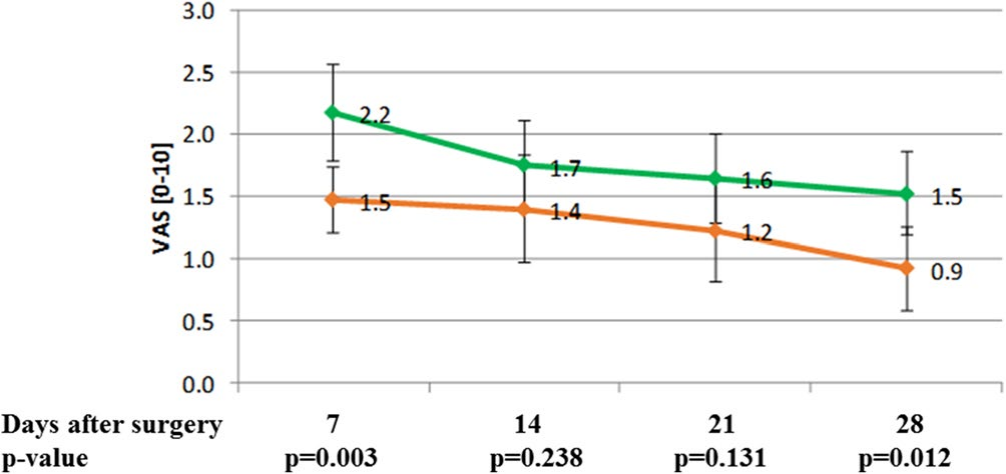
Pain in patients with higher and lower dose of tramadol with paracetamol during the first 28 days after axillary lymphadenectomy (red line—higher dose group, green line lower dose group).

Pain in our patients was from 0 VAS score to 7 VAS score during the first 4 weeks after surgery. A mean VAS value for pain was 1.8, 1.6, 1.4 and 1.2 on the 7th, 14th, 21st and 28th days after surgery, respectively. Patients with the higher dose of tramadol had statistically significantly less pain during the 1st (p = 0.003) and 4th week (p = 0.012) than patients with the lower dose (Fig. 2).

### Side effects

At least one side effect was experienced by 88% of patients. Any side effect was present in 86%, 62%, 50% and 43% of patients during the 1st, 2nd, 3rd and 4th weeks, respectively. Among all patients, 36% experienced nausea, 9% vomiting, 35% dizziness and 46% constipation. Physicians estimated that there was a causal connection between treatment with tramadol and patients’ side effects in 88% of cases.

**Figure 3:**
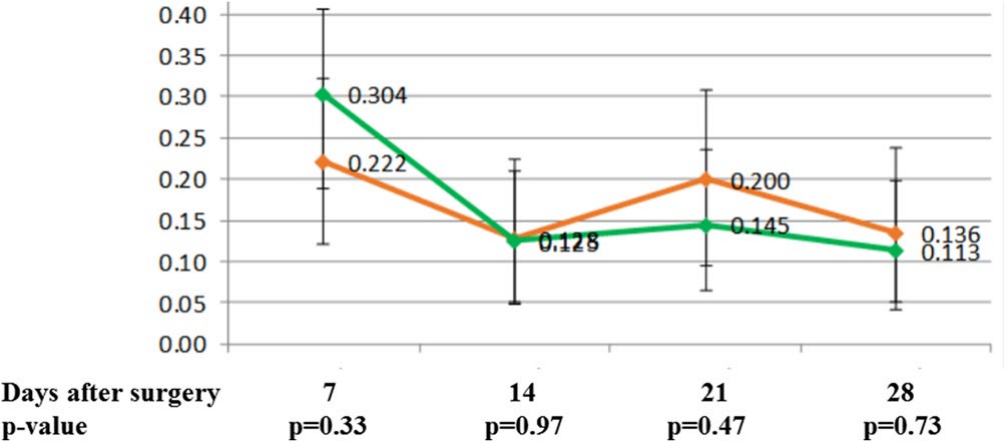
Nausea in patients with higher and lower dose of tramadol with paracetamol during the first 28 days after axillary lymphadenectomy (red line—higher dose group, green line lower dose group).

**Figure 4:**
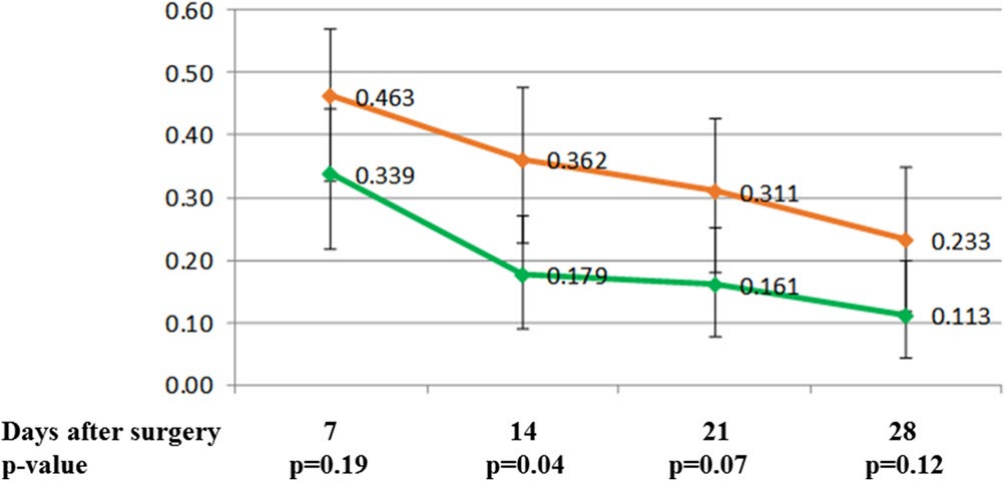
Constipation in patients with higher and lower dose of tramadol with paracetamol during the first 28 days after axillary lymphadenectomy (red line—higher dose group, green line lower dose group).

Frequency of nausea and vomiting (Fig. 3) were not significantly different between the two treatment groups of patients. Nausea was present in 26%, 12%, 17% and 12% of patients during the 1st, 2nd, 3rd and 4th weeks, respectively. Vomiting was present in 6%, 1%, 5% and 4% of patients during the 1st, 2nd, 3rd and 4th weeks, respectively. Dizziness was present in 30%, 11%, 9% and 10% of patients during the 1st, 2nd, 3rd and 4th weeks, respectively. Constipation was present in 40%, 26%, 23% and 17% of patients during the 1st, 2nd, 3rd and 4th weeks, respectively. Constipation was significantly more common in the group with stronger analgesia during the 2nd week (p = 0.04) in comparison to patients with weaker analgesia (Fig. 4).

Dry mouth was reported in 25% of patients, somnolence in 22.5%, flatulence in 8%, sweating in 8%, mood change in 6%, headache in 5%, pruritus in 4%, sleep disorder in 3%, abdominal pain in 2.5%, tremor in 2.5%, hypertension in 1.7%, allergic reaction in 0.8%, confusion in 0.8%, diarrhea in 0.8%, dyspepsia in 0.8% and bitter taste in 0.8%.

### Lymphedema and shoulder movement

None of our patients had lymphedema 1 month after surgical procedure. Range of shoulder movement was not significantly different between the two treatment groups of patients. Mean shoulder movement was 125°, 140°, 145° and 149° during the 1st, 2nd, 3rd and 4th weeks, respectively.

### Patients who abandoned the study

Treatment with the test drugs was discontinued in 15 patients. One patient had an allergic skin reaction immediately after ingestion of the first dose of the analgesic test drug, so she discontinued the treatment with tramadol. Another 14 patients discontinued treatment with the test drugs due to adverse side effects. Patients who abandoned the study protocol had nausea, dizziness, constipation and vomiting in 10, 4, 4 and 2 cases, respectively.

## Discussion

Axillary surgery in breast cancer patients may have a deteriorating effect on arm morbidity, movement limitations, swelling and lymph drainage^4^. Risk factors for chronic persistent pain are younger age, radiotherapy, axillary lymph node dissection and greater acute postoperative pain^12^. Greater acute postoperative pain has an odds ratio of 1.16 for peristent pain for every one point in the 10 point VAS scale, as shown by a meta-analysis of observational studies reported by Wang et al.^12^. However, a systematic review of observational studies has found no compelling evidence to support the prevention of persistent postsurgical pain by perioperative pain treatment after breast cancer surgery^12^. To our knowledge, there are only limited data on interventional studies about the treatment of acute pain after breast cancer surgery. The aim of our intervention double-blind randomized study was to evaluate the severity of acute pain and the frequency of side effects in breast cancer patients treated with two regimens of tramadol with paracetamol after axillary lymphadenectomy. We found that the patients with the higher dose of tramadol with paracetamol had less pain during the 1st and 4th weeks than the patients with the lower dose. Lymphedema or range of shoulder movement was not significantly different between the two groups of patients during the first 29 days.

Ten days after the modified radical mastectomy Amr and Yousef^13^ reported, the VAS score at rest between 0.2 and 2.4 and during movement between 1.8 and 3.7 in the patients who were on acetaminophen 500 mg/codeine 30 mg tablets every 6 h. Similarly, after a modified radical mastectomy or quadrantectomy with axillary lymphadenectomy Reyad et al.^13^ reported, the mean VAS score 2.2 (± 1.1), 2.4 (± 1.3), and 2.7 (± 1.7) after 7, 14, and 21 days, respectively. Patients of Reyad et al.^13^ had pain killers, but medications were taken at the patient's discretion as needed. On the other hand, both our groups of patients had low mean VAS scores. This is understandable, as all our patients took analgesics regularly at prescribed doses and intervals. In addition to taking analgesics on a regular basis, the low mean VAS score also allowed for regular shoulder girdle mobility exercises. It is obvious that regular use of medication in the postoperative period in the appropriate dose leads to a reduction in acute pain. The intensity of acute pain, however, affects the frequency of late sequelae. Despite small absolute difference in the intensity of pain between our two groups of patients, those patients who received a stronger postoperative analgesia had less arm symptoms and a better quality of life 1 year after surgery in comparison to patients who received a weaker analgesia^11^. Furthermore, the patients who received a stronger postoperative analgesia had a statistical trend for less neuropathic pain in comparison to patients who received a weaker analgesia^11^.

The appropriate treatment of acute pain is associated with the prevention of persistent pain after an axillary lymphadenectomy, as shown by a meta-analysis involving a total of 19,813 patients^12^. Similarly to Reyad et al.^14^, we have observed that patients have frequently pain in the 2nd and 3rd week after surgery. During this period shoulder movement becomes very painful due to the fibrous scar that limits a successful physical therapy^11^. In one of our recent publications it was already reported^11^, that there was a statistical correlation between more limited shoulder movement 1 year after surgery and greater pain on the 2nd postoperative day, 2 weeks after surgery, 3 weeks after surgery and 4 weeks after surgery^11^. Therefore, effective analgesia is crucial in order to prevent limited long lasting shoulder movement. After surgical procedure we had to convince the patients that they have to use analgesics and perform shoulder girdle mobility exercises on a regular basis and thus prevent chronic problems. We needed to tell the patient that she will not do any harm to herself by taking analgesics and exercising regularly.

Use of opioids is frequently limited by their side effects^10^. Tramadol was used in our study because it produces satisfactory analgesia against various types of pain, and it is currently approved for the treatment of moderate to severe pain^9^. Furthermore, abuse and dependence are rare in tramadol users^15^. It was reported that tramadol induces fewer side effects than classic opioids^9^. But tramadol was discontinued due to adverse events in 15 (12.8%) of our patients. Furthermore, 88% of patients experienced at least one of the side effects. The most common side effects in our patients were constipation, nausea and dizziness, which were experienced in 46%, 36% and 35% of patients, respectively. Our observations are consistent with data from the literature. In a review article, Vazzana et al.^10^ published that the most commonly reported adverse events from the therapeutic use of tramadol included dizziness, followed by vomiting, nausea, somnolence and constipation.

The occurrence and severity of side effects is related to the dose of opioids^10,16^. DeLemos et al.^16^, in a randomized, double-blind, placebo-controlled multiple-ascending-dose study, administered 200, 400, or 600 mg/day tramadol to 21 healthy adults. Their participants reported ≥ 1 treatment-emergent adverse event in 87.5% of cases, and the most frequent were nausea (71%) and vomiting (29%)^16^. Vomiting increased with an increasing dose from 200 to 600 mg/day, but was only mild (5 of 24 cases) or moderate (2 of 24 cases) in severity^16^. DeLemos et al.^16^ concluded that all tested dosage regimens of tramadol showed an acceptable safety and tolerability profile. On the other side, our patients received only 112.5 or 225 mg/day, so their side effects were less common in comparison to the former study. It has been known for a long time that a slower titration rate of tramadol improves tolerability in patients, since it affects nausea and/or vomiting^17^. Frequency of nausea or vomiting was not significantly different between the two groups of our patients. However, the vast majority of our patients who discontinued use of tramadol were from the higher dose group (p = 0.004). The only statistically significant difference between the higher and lower dose groups of patients with regard to side effects was constipation. Constipation was significantly more common only during the 2nd week in the group with stronger analgesia in comparison to patients with weaker analgesia. Because there was no difference in really unpleasant side effects (nausea and vomiting), and patients on the higher dose of analgesia had less pain than patients on the lower dose of analgesia, we advocate the higher dose of analgesia for treatment of breast cancer patients after axillary lymphadenectomy.

Of course, our study has several limitations. It included a relatively small number of patients. Furthermore, pain in both treatment groups of patients was less severe than expected and observed in our historic series (unpublished data). One possible reason might be the use of a continuous local infusion of 0.25% levo-bupivacaine with a fenestrated wound catheter in all patients. Another possible reason might be that the patients had to fill out the diary regularly, and were closely monitored for regular use of analgesics and shoulder exercises. But we believe that keeping a diary about side effects three times a day is probably controversial and resulted in over-reported side effects. Physicians in the outpatients’ clinic often had the impression that our patients wanted to please us, and reported as much as possible during interviews about the side effects, despite there being no objective signs of side effects. Therefore we believe that side effects should not be mentioned in diary form for patients. But regular use of analgesics is very beneficial, and we believe that it should be recorded in diary form in order to improve compliance with regular use of analgesics and shoulder exercises.

We observed that some of our patients experienced insufficient pain relief or adverse events. One way to identify patients with insufficient effect or intolerance to tramadol would be pharmacogenomics testing, but currently there is a lack of convincing evidence to support pharmacogenomics testing when initiating opioid therapy^18^. CYP2D6 is the main metabolizing enzyme of tramadol, leading to the formation of the metabolite with the highest analgesic activity^19^. Genetic variability in the highly polymorphic CYP2D6 gene could therefore contribute to differences in response to tramadol. The CYP2D6 phenotype was significantly associated only with constipation, but none of the CYP2D6 poor metabolizers experienced constipation or vomiting^20^. Because genetic variability of genes involved in tramadol pharmacokinetics may be associated with adverse events in breast cancer patients, we believe that pharmacogenetic testing could enable a more personalized treatment.

## Conclusions

The patients who received tramadol with paracetamol at a dose of 75/650 mg had less pain in comparison to patients who received tramadol with paracetamol at a dose of 37.5/325 mg. Side effects were mild, but common in both groups of patients, and constipation was the only side effect that was more common, for 1 week only, in patients on the higher dose of tramadol.
